# Effectiveness of the Semi‐Automated Post‐ANaesthesia Discharge Assessment Tool: A Pre‐Post Study Using Propensity Score Matching

**DOI:** 10.1111/nicc.70393

**Published:** 2026-02-11

**Authors:** Benjamin Albiez, Jan Breckwoldt, Julia Braun, Hannele Hediger, Michael Tucci, Donat R. Spahn, Sonja Beckmann

**Affiliations:** ^1^ Institute of Anaesthesiology University and University Hospital Zurich Zurich Switzerland; ^2^ Departement of Health Profession Zurich University of Applied Science Winterthur Switzerland; ^3^ Department of Epidemiology and Biostatistics, Epidemiology, Biostatistics and Prevention Institute University of Zurich Zurich Switzerland; ^4^ Centre of Clinical Nursing Science University Hospital Zurich Zurich Switzerland

**Keywords:** discharge assessment, discharge confidence, discharge efficiency, length of stay, operating room hold, postanaesthesia care unit

## Abstract

**Background:**

Discharge management and workflow efficiency in a postanaesthesia care unit (PACU) can be improved with specific tools assessing discharge readiness. Several authors have found tools to reduce the PACU length of stay (PACU LOS), but results have remained inconsistent.

**Aim:**

We analysed the effects of the Post‐ANaesthesia Discharge Assessment tool (PANDA) on PACU LOS, operating room (OR) holds, PACU nurses' confidence with the discharge decision and their perception of the tools' implementation.

**Study Design:**

This pre–post study with a propensity‐matched historical control group evaluated the impact of the semi‐automatic PANDA tool in a single primary‐level hospital. The tool supports discharge decisions in the PACU. Median PACU LOS pre‐ and post‐implementation was compared using nearest‐neighbour propensity score matching and weighted linear regression. OR holds were analysed over 20 consecutive days. A structured nurse survey assessed confidence in discharge decisions and perceptions of the tool's implementation.

**Results:**

The study included 8475 patients (pre *n* = 4509; post *n* = 3966) and 19 nurses. Median PACU LOS before implementing the PANDA tool was 114 min (IQR 89–144) compared to 103 min (IQR 79–136) after the implementation. The weighted linear model showed an estimated difference in PACU LOS of −16 min (95% CI from −20 to −12 min, *p* < 0.001). There were too few OR holds for comparison. PACU nurses' confidence in their discharge decision remained unchanged before and after implementation. The PANDA tool received high ratings for acceptability, appropriateness, compatibility and feasibility.

**Conclusion:**

Implementing the semi‐automated PANDA discharge tool significantly decreased PACU LOS. In addition, PACU nurses reported high acceptance, usefulness and feasibility of the tool. The PANDA discharge tool may optimise routine clinical practice to streamline PACU workflows, support resource allocation and decision‐making and promote standardisation. Reducing PACU LOS may also improve patient flow and capacity planning in high‐volume settings.

**Relevance to Clinical Practice:**

The semi‐automated PANDA tool was well received by nurses who perceived it as useful and feasible. Given the significant reduction in PACU length of stay, integrating PANDA into clinical practice may enhance post‐surgical patient flow and resource allocation, while its main added value lies in improving standardisation, supporting decision‐making and PACU workflow.

## Impact Statement

- What is known about this topic?
  ○ Timely discharge from the postanaesthesia care unit (PACU) is essential for optimising patient flow and efficient use of resources.
  ○ Structured discharge tools have shown potential to reduce PACU length of stay (LOS), though previous studies were often limited by small samples, inadequate controls or methodological bias.
  ○ Additional documentation burdens may hinder tool adherence by nurses, highlighting the need for systems that integrate into existing clinical workflows.
- What this paper adds?
  ○ This study showed that a semi‐automated discharge tool (PANDA), which draws on routinely documented electronic patient data, significantly reduced PACU LOS in a large pre–post analysis using propensity score matching.
  ○ PACU nurses reported high levels of acceptance, usefulness and feasibility, indicating strong potential for sustainable implementation.
  ○ PANDA's main added value lies in improving standardisation, supporting decision‐making, and PACU workflow.

## 1 Introduction

Nurses in the postanaesthesia care unit (PACU) must decide whether post‐surgery patients are ready for discharge to a facility with less frequent and less invasive monitoring of vital parameters [1]. A timely discharge is desirable to optimise the use of PACU facilities and personnel. In addition, timely discharge ensures free patient inflow from the operating room (OR). An OR hold occurs when a fully occupied PACU inhibits the transfer of a patient after surgery, requiring the anaesthesia team to continue caring for the patient within the operating room and potentially leading to delays of subsequent procedures.

The use of structured tools to determine discharge readiness may improve nursing management and increase workflow efficiency [2]. Several studies have compared discharge assessment tools against various controls (e.g., physician‐directed discharge, time‐based discharge or other discharge tools) [3, 4, 5, 6]. In general, the authors found the tools to be safe and appropriate for identifying the discharge readiness of PACU patients [2, 3, 4, 5, 7]. Moreover, most papers reported that the introduction of a discharge tool decreased PACU length of stay (LOS) by between 4.5 and 31.4 min [5, 7]. However, the value of previous studies is limited by several shortcomings, including inadequate control groups, insufficient sample sizes or high risks of selection bias [2, 3, 4, 5, 6]. Therefore, it remains unclear whether such decision tools benefit PACU LOS.

One potential explanation for the varying levels of effectiveness of discharge tools could be the additional documentation and record keeping involved, which may be seen as an extra burden by nurses. A lack of adherence may lead to delayed decisions and [7, 8] more importantly, insufficient record keeping may raise medico‐legal concerns [8, 9]. To address these concerns, it would be helpful to retrieve as many parameters automatically from the patient record and thereby minimise the additional work for PACU nurses. The vital parameters recorded by electronic patient data management systems (PDMS) are easily available and could be used to produce a score, thus relieving the staff and leaving only a few parameters which must be assessed ‘by hand’ (e.g., pain intensity) and entered into the system.

In this study, we examined the impact of a specifically developed, semi‐automated discharge assessment tool. Ideally, this would have been assessed in a randomised trial; however, this was not possible for organisational reasons. It was therefore important to use a statistical design that was as robust as possible in the given situation. We chose propensity score matching as a valid, albeit second‐best, alternative when a randomised trial is not possible.

For a sufficient sample size and high‐quality data, we used the patient records generated by the PDMS. Another important point was to retrieve data on the PACU nurses' confidence with the discharge decision and nurses' satisfaction with the tool, thereby controlling for potential negative side effects of implementation.

### 1.1 Aim and Objectives for the Study

The primary outcome variable was the median PACU LOS difference between the pre‐ and post‐implementation periods. Our secondary outcomes were differences in nurses' confidence with the discharge decision and satisfaction with the tool. We also analysed the difference in OR holds, and differences between surgical disciplines.

### 1.2 Research Question

Does the implementation of a semi‐automated discharge assessment tool (PANDA) reduce the median length of stay in the PACU, and what effect does it have on the frequency of OR holds, as well as on nurses' confidence in discharge decisions and their satisfaction with the tool?

## 2 Design and Methods

### 2.1 Setting and Sample

This single‐centre, pre–post study with a propensity‐matched historical control group was conducted in the PACU of a primary‐level hospital. This is a tertiary academic centre with 900 beds serving 43 medical specialties including the entire spectrum of surgery, except elective orthopaedic and paediatric surgery. Annually, more than 30 000 surgical interventions with anaesthesia are performed in 34 ORs at our hospital. Around 20 000 of these patients are treated in one of the four PACU sites resulting in a daily average of 60 patients. A team of 39 PACU nurses serve a total of 27 beds, with a patient to nurse ratio of 3:1. The former discharge process at the institution combined physician‐directed and time‐based strategies without having defined a specific time frame.

### 2.2 The Post‐ANaesthesia Discharge Assessment (PANDA) Tool

After a literature review on PACU discharge assessment tools, the author team identified the ‘Readiness for Discharge Assessment Tool’ (RDAT) as the most appropriate and feasible starting point [4]. The instrument includes 10 items to be answered with ‘yes’ or ‘no’ and has excellent psychometric properties (content validity index 0.80, Cronbach's alpha = 1.0 for interrater reliability) [4]. The instrument was translated from English to German and backwards following established guidelines for the translation of scientific texts [10]. Afterwards, we adapted the tool to the German‐speaking Swiss context using a modified Delphi process [11, 12].

Our Delphi group consisted of 26 nurses and physicians from the anaesthesia and PACU of our institution and two other major Swiss hospitals. The group discussed and revised the tool in accordance with the current best practice guidelines until consensus was reached. For example, we removed the ‘body temperature’ item from the tool because in Switzerland normothermia is mandatory for a patient to be transferred from the operating room to the PACU [4]. All changes to the tool were discussed with and approved by the developers of the original RDAT tool.

**FIGURE 1:**
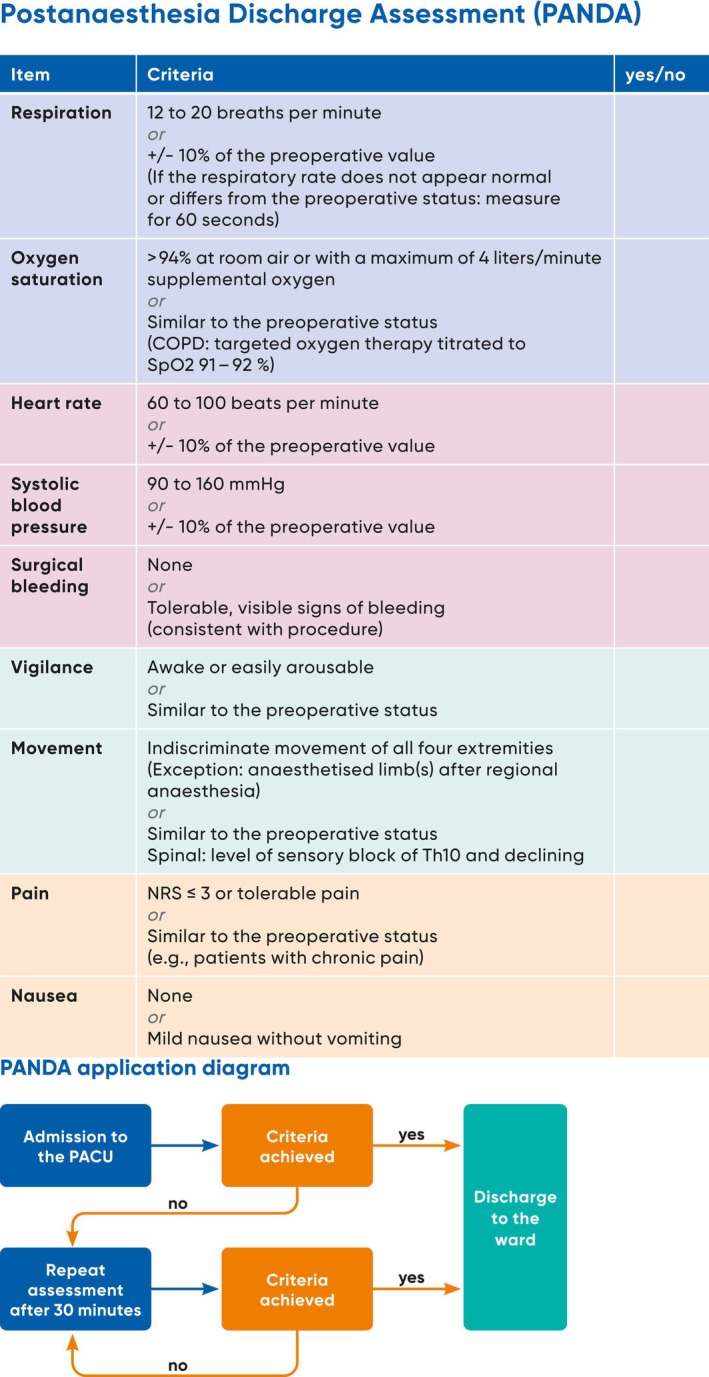
The post‐anaesthesia discharge assessment (PANDA) tool has nine items, which are assessed dichotomously with ‘yes’ or ‘no’. Discharge readiness is achieved when nine items are rated ‘yes’. The PANDA is an instrument to assess discharge readiness of postsurgical patients from the PACU to the ward. The instrument contains nine clinical items with associated criteria, and is applicable to adult patients after general or regional anaesthesia. Cardiac or neurosurgical patients, patients with a caesarean section, or prolonged recovery and overnight patients cannot be assessed with the PANDA. To achieve discharge readiness to the ward, all items must be rated with ‘yes’. After 150 min or five assessments without achievement of discharge readiness a physician anaesthestist must be involved for further direction.

The final 9‐item ‘Post‐ANaesthesia Discharge Assessment’ (PANDA) tool (see Figure 1) was integrated into the hospital wide electronic PDMS as a semi‐automatic assessment. The vital parameters oxygen saturation, respiration rate, heart rate and systolic blood pressure were automatically obtained from the PDMS and rated as ‘yes’ or ‘no’ with regards to falling within predefined limits. To generate the total PANDA score, PACU nurses validated each value in the PDMS and added the qualitative information for pain, mobility/activity, surgical bleeding, vigilance/mental status and nausea/vomiting. Patients can only be discharged if they meet all criteria, achieving a PANDA score of 9 (see Figure S1). The first assessment was to be completed at PACU admission and then repeated every 30 min until discharge. Nurses received a visual reminder every 30 min by a push‐message on the PDMS screen until all nine items were rated ‘yes’ and the patient reached discharge readiness. The 30‐min interval was chosen to keep the workload acceptable [13, 14, 15]. If discharge readiness was not achieved within 150 min, a physician anaesthetist assessed the patient for further directives.

In September 2021, all PACU nurses were trained to use the PANDA tool and starting on 4 October 2021, the PACU discharge of all adult patients (age ≥ 18 years) was supported by PANDA. Some patients were not treated on PACUs due to different clinical pathways, including patients who were directly transferred to intensive care or intermediate care units, such as in major neurosurgery (e.g., Craniotomy) and major cardiac surgery (e.g., on‐pump surgery); obstetric patients after caesarean section who are directly re‐transferred to the delivery area; and patients with peripheral nerve block who are directly transferred to the ward.

### 2.3 Data Collection, Tools and Methods

PACU LOS was defined as the time in minutes between admittance and discharge from the PACU. Anonymised patient data for the propensity‐matched historical control group were retrieved from the PDMS for two different time periods: from 6 January 2020 until 3 October 2021 (pre‐implementation, control group) and from 4 October 2021 until 31 January 2022 (post‐implementation, PANDA group). To ensure an adequate amount of data for matching, we opted for oversampling during the pre‐implementation phase. Patient data from the pre‐ and post‐implementation periods were compared by propensity score matching, and further details are provided below.

An OR hold was defined as an event when a patient could not be transferred from the OR area to the PACU due to PACU occupation. The number of events and duration in minutes were documented in all four PACUs before and after the implementation of PANDA. The analysis of OR holds was limited to a 20‐day period because the timeframe for data collection was constrained and needed to align with operational feasibility; therefore, we selected a continuous interval representative of routine PACU workflow (Monday to Friday) from 7 am to 4 pm (control group: 1–31 August 2021, PANDA group: 25 October to 19 November 2021). Data were routinely controlled for completeness by the first author (BA). Patient data and demographic characteristics from nurses were collected from nurses and medical.

Online surveys assessed PACU nurses' *confidence with the discharge decision* and the *perception of the implementation* of the PANDA. The survey was developed by the research team and piloted with three experienced PACU nurses from the same hospital to ensure clarity. *Confidence with the discharge decision* was rated on a slide bar ranging from 0 (no confidence) to 100 (full confidence). The *perception of the implementation* was assessed with five questions on acceptability, usability, compatibility, appropriateness and feasibility of the tool. Items were answered on a 4‐point Likert‐like scale, ranging from 4 (agree) to 1 (disagree) [16]. In addition, information was collected about participants' age, gender, education, experience in nursing and experience in the PACU work.

Data collection was before and after the implementation (August and December 2021, respectively). Although confidence with the discharge decision was assessed at both timepoints, *perception of the implementation* was only assessed after the implementation. All PACU nursing staff were invited to participate in the two anonymous, voluntary online surveys via Redcap [17], and they received two reminders. Participants provided electronic informed consent before completing the questionnaire.

### 2.4 Data Analysis

For continuous variables, we show means and standard deviations (SD) along with median, 25th and 75th quantile, interquartile range (IQR), minimum, maximum and number of missing values. For categorical variables, we show frequencies and percentages along with the number of missings.

PACU LOS was compared in the control and PANDA group using nearest‐neighbour propensity score matching with replacement. The propensity scores were calculated using the covariates: gender, age, ‘American Society of Anesthesia physical status’ (ASA‐PS) classification, type of surgery and duration of anaesthesia. To assess whether an acceptable balance in the matched groups was achieved, absolute standardised mean differences between the two obtained groups were calculated for all variables included in the propensity score before and after matching. If these values are below 0.1, good balance of the two groups has been reached [18, 19]. Using a matching algorithm with replacement means that more balanced groups can be reached, but it also means that a weighting scheme must be used in the analyses to make sure that persons who occur in the data set more than once can be treated correctly [20]. The duration of PACU LOS in the two matched groups was therefore compared using a weighted linear model. A sub‐analysis examined PACU LOS according to the surgical discipline by adding the surgical discipline to the linear model, setting plastic surgery as the reference category. In order to conduct comparisons of the two groups within each discipline, an interaction term between the two variables was added to the final model.

Nurses' confidence with the discharge decision before and after implementation was compared using a Wilcoxon test. Data were analysed using the statistics programme R (Version 4.0.5) [21]. The alpha level was set at *p* < 0.05 [22].

### 2.5 Ethical Approvals

The study was approved by the Ethics Committee and issued a declaration of no objection (Request number: 2021‐00806, on 20 July 2021, Chairperson: P. Kleist). The reporting of this study adheres to the STROBE guidelines.

## 3 Results

### 3.1 PACU LOS

**FIGURE 2:**
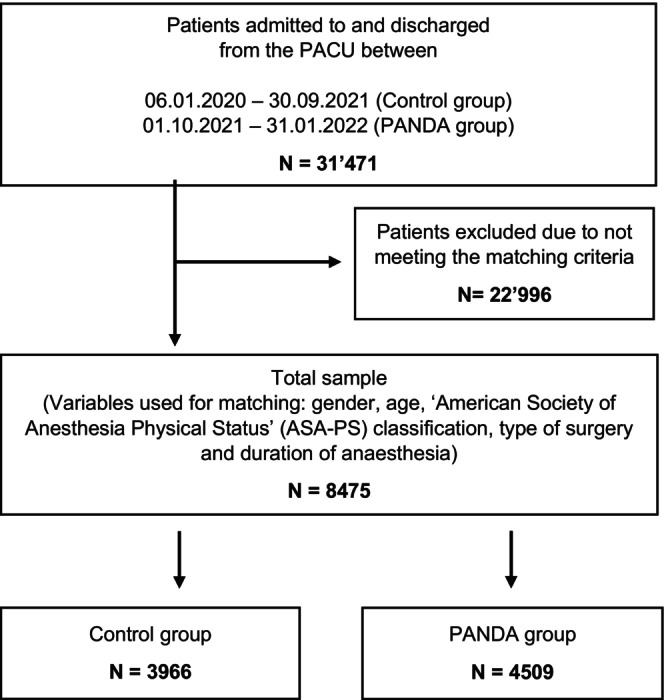
Flowchart of the propensity score matching process with replacement. The matching criteria were gender, age, American Society of Anaesthesiologists (ASA) classification, type of surgery and duration of anaesthesia. Individuals of the control group (*n* = 3966) were matched as appropriate: *N* = 3487 were matched once to the PANDA group, *n* = 425 were matched twice, *n* = 47 were matched three times, *n* = 6 were matched four times, and one person was matched five times. The control group reached *n* = 4509 with replacement. PANDA, postanaesthesia discharge assessment; PACU, post anaesthesia care unit.

Datasets of 31 469 patients were available for analysis of which 8475 met the matching criteria. The PANDA group (*n* = 4509) was matched to a control group of 3966 patients, reaching *n* = 4509 due to replacement (see Figure 2). In the control group, *n* = 3487 patients matched once, *n* = 425 matched twice, and *n* = 47 matched 3 times, *n* = 6 matched 4 times and 1 patient matched 5 times. This is reflected in the weights used in the regression analysis. Table 1 shows the patient characteristics. Regarding the propensity score matching, the two groups had balanced standardised mean differences for all variables: age, gender, ASA classification, type of surgery and duration of anaesthesia (see Figure S2).

**TABLE 1:** Patient characteristics of the propensity score matched groups before (control group) and after (PANDA group) the implementation of the PANDA.

Characteristics	Control group	PANDA group
Sample size, *n*	3966	4509
Age, in years, mean (±SD)	53.3 (18.6)	53.4 (18.1)
Gender, *n* (%)		
Male	1866 (47.0)	2200 (48.8)
Female	2100 (53.0)	2307 (51.2)
ASA score, *n* (%)		
1	393 (9.9)	428 (9.5)
2	1856 (46.8)	2099 (46.6)
3	1488 (37.5)	1721 (38.2)
4	228 (5.8)	257 (5.7)
5	1 (0.0)	2 (0.0)
Duration of anaesthesia, in minutes, median (IQR)	145 (106–204)	147 (110–198)
Duration of surgery, in minutes, median (IQR)	55 (29–101)	55 (29–99)
Type of anaesthesia, *n* (%)		
General (Volatile)	2128 (53.7)	2501 (55.5)
General (TIVA)	1614 (40.7)	1859 (41.2)
Regional Isolated	66 (1.7)	44 (0.1)
Others	158 (4.1)	103 (2.2)
Type of surgery, *n* (%)		
Plastic surgery	537 (13.5)	676 (15.0)
Neurosurgery	95 (2.4)	108 (2.4)
Oral maxillofacial surgery	173 (4.4)	298 (4.6)
Ophthalmic surgery	259 (6.5)	285 (6.3)
Ear, nose, throat, pharynx surgery	797 (20.1)	894 (19.8)
Thoracic surgery	59 (1.5)	52 (1.1)
Cardiac/vascular surgery	140 (3.5)	137 (3.0)
General and bariatric surgery	459 (11.6)	528 (11.7)
Urologic surgery	383 (9.7)	421 (9.3)
Gynaecologic surgery	634 (16.0)	688 (15.3)
Trauma/orthopaedic surgery	396 (10.0)	475 (10.5)
Transplantation surgery	18 (0.5)	12 (0.3)
Other	16 (0.4)	23 (0.5)

Abbreviations: ASA, American Society of Anaesthesiologists; IQR, interquartile range; PANDA, post‐anaesthesia discharge assessment; SD, standard deviation; TIVA, total intravenous anaesthesia.

**FIGURE 3:**
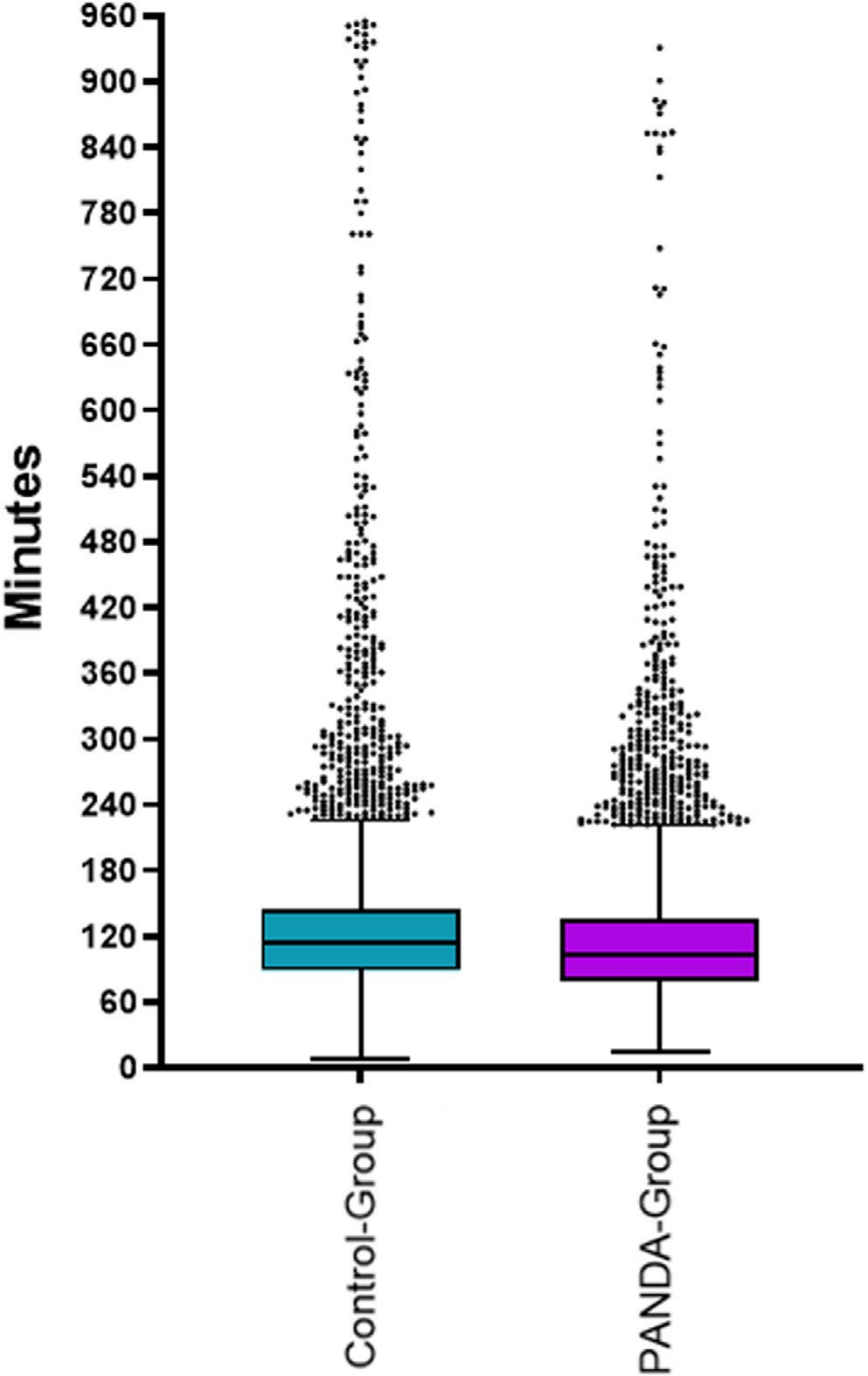
The postanaesthesia care unit length of stay (PACU LOS) time difference between the control and PANDA group. The boxplot with median, interquartile range (IQR) and Whisker length 1.5 × IQR and outliers shown as staggered points. PACU LOS in the control group was median 103 min (IQR 79–136), compared to 114 min (IQR 89–144) in the PANDA group.

The median PACU LOS in the control group was 114 min (IQR 89–144) compared to the PANDA group with 103 min (IQR 79–136) (see Figure 3). The weighted linear model showed an estimated difference in PACU LOS between the two groups of −16 min (95% CI from −20 to −12 min, *p* < 0.001) (see Table S1). Five hundred eighty four patients (13%) did not achieve the full PANDA score for discharge readiness within 150 min. These patients were discharged by a physician anaesthetist.

Stratified for surgical disciplines, the implementation of the PANDA tool reduced PACU LOS in all surgical disciplines (Table 2). We found significant time reduction in general, cardiac, neuro, kidney transplantation and other surgeries, but not in ophthalmic surgery, urology and gynaecology. Table 2 also shows the raw data of the control group before matching, indicating that the reduction in PACU LOS trends in the same direction, even without matching.

**TABLE 2:** Median (IQR) PACU duration of the PANDA and the control group (before and after matching) according to the surgical discipline.

Surgical discipline	PANDA group		Control group after matching		*p*	Control group before matching	
	*n*	PACU duration in minutes median (IQR)	*n*	PACU duration in minutes median (IQR)		*n*	PACU duration in minutes median (IQR)
Ophthalmic surgery	285	93 (76–119)	259	101 (73–122)	0.26	3198	89 (57–114)
Urologic surgery	421	86 (69–111)	383	106 (86–130)	1.0	2391	106 (86–132)
Ear, nose, throat, pharynx surgery	894	97 (79–125)	797	108 (90–132)	0.95	4500	107 (89–130)
Gynaecologic surgery	688	94 (72–124)	634	108 (86.2–133.8)	1.00	4156	107 (89–130)
Oral maxillofacial surgery	208	111 (86–144)	173	118 (98–138)	0.90	1067	117 (99–140.5)
Plastic surgery	676	105 (82–138)	537	115 (91–141)	0.23	3461	115 (94–141)
Thoracic surgery	52	121.5 (91–176.8)	59	123 (98–157)	1.0	299	123 (95.5–165.5)
Trauma/orthopaedic surgery	475	117 (88.5–161)	396	123.5 (99.8–160.5)	0.4	3008	121 (93–160)
General and bariatric surgery	528	114 (89–167)	459	129 (99–176.5)	0.02*	2841	125 (98–167)
Cardiac/vascular surgery	137	111 (82–159)	140	127.5 (98.5–184)	0.02*	874	129 (96–178)
Neurosurgery	108	125.5 (102–181.8)	95	136 (92–197.5)	0.02*	815	131 (97–190.5)
Kidney transplantation surgery	12	166.5 (135.5–293.8)	18	414 (173.2–522.8)	< 0.0001*	117	347 (114–507)
Other	23	116 (98–177)	16	221 (95.8–452.8)	< 0.0001*	235	166 (102.5–338.5)
Total	4509	103 (79–136)	3966	114 (89–144)	< 0.001*	26 962	111 (87–140)

*Note:* The PANDA and the control group after matching were compared with a weighted linear model, using an interaction term between surgery type and group.

### 3.2 OR Holds

Overall, we noted only six OR holds during the study period: four OR holds with a total duration of 90 min in the control group (*n* = 969), and 2 OR holds with a total duration of 50 min in the PANDA group (*n* = 1090). We did not perform any statistical analysis because of the small number of events.

### 3.3 PACU Nurses' Confidence With the Discharge Decision

In each survey before and after the implementation, 19 out of 39 eligible PACU nurses participated (48%). As participation was anonymous, it remains unclear whether or not the samples are made up of the same participants. Table 3 shows the characteristics of the PACU nurses and confidence with the discharge decision values. Median confidence in the pre‐implementation period was 97 (IQR 87.8–99) on a scale from 0 to 100. Post‐implementation of the median confidence was 95 (IQR 90–98.5). We could not show a statistical difference between the two periods (*p* = 0.845).

**TABLE 3:** Characteristics of PACU nurses and their confidence with the discharge decision.

Characteristics	Control group	PANDA group
Sample size, *n*	19	19
Age, in years, mean (SD)	49.2 (9.5)	46.2 (11.3)
Gender, *n* (%)		
Male	2 (10.5)	2 (10.5)
Female	17 (89.5)	17 (89.5)
Education		
Registered nurse, *n* (%)	5 (26.3)	5 (26.3)
Registered nurse with intermediate care course, *n* (%)	8 (42.1)	8 (42.1)
Registered nurse + anaesthesia diploma, *n* (%)	1 (5.3)	1 (5.3)
Registered nurse + intensive care diploma, *n* (%)	3 (15.3)	3 (15.3)
Other, *n* (%)	2 (10.5)	2 (10.5)
Experience in Nursing, in years, mean (SD)	23.2 (8.8)	23.2 (8.8)
Experience in PACU, in years, mean (SD)	9.9 (6.7)	9.8 (6.7)
Confidence with discharge decision, median (IQR)	97 (87.8–99.0)	95 (90.0–98.5)

*Note:* The confidence scale ranges from 0 (no confidence) to 100 (full confidence).

Abbreviations: PANDA, postanaesthesia discharge assessment, PACU, postanaesthesia care unit; SD, standard deviation; IQR, interquartile range.

### 3.4 PACU Nurses' Perception of the Implementation of the PANDA Tool

The implementation of the PANDA tool was well received by all respondents. Sixteen respondents (84%) rated PANDA as an acceptable tool in clinical practice, 19 participants (100%) found it simple to use, 16 (84%) found it feasible and appropriate to assess discharge readiness, and 18 (95%) respondents (95%) found the tool compatible with the work in the PACU.

## 4 Discussion

This study showed that the semi‐automated discharge tool PANDA significantly reduced PACU LOS. The median decrease of 11 min (and 16 min based on the weighted linear regression model) translates to more than 1800 working hours per 10 000 PACU patients (typical number of patients per year in a medium sized hospital). Although the reduction of 16 min is modest, it may still meaningfully improve PACU workflow fluidity in high‐volume settings and help reduce bottlenecks. However, the primary justification for implementing the tool lies not only in potential timesavings but also in the enhanced standardisation and decision‐making support it provides. Our results confirm findings from previous studies, which showed that discharge assessment tools decreased PACU LOS more effectively than other discharge strategies [23, 24, 25, 26]. However, our study analysed a much larger sample than previous studies and applied a far more rigid methodology. By using propensity score matching, we applied the best method possible to create comparable groups if randomisation is not possible. Because the control period overlapped with the COVID‐19 pandemic, pandemic‐related operational adjustments may have influenced OR activity and PACU workflows. However, using propensity score matching as the analysis method for the group comparisons should have reduced potential systematic differences. As we adapted the original RDAT tool to the Swiss setting and the current guidelines, we recommend that the psychometric parameters should be examined in a future study.

As a second important point, PACU nurses perceived the tool as feasible, useful and supportive. This was not necessarily expected as the implementation of a new system might increase workload. In addition, nurses could have perceived an automated tool as a limitation of their professional autonomy and might have developed psychological resistance [8]. Resistance to change often arises from individual (e.g., fear, uncertainty), interpersonal (e.g., communication, peer norms) and organisational factors (e.g., workload, lack of involvement in decision‐making, insufficient training). Our results therefore suggest that these potential barriers were successfully addressed by the implementation strategy involving staff and clear communication as supportive. This success may serve as an example for other institutions planning to introduce automated workflows in the PACU [27].

As one possible explanation for the high acceptance, the interprofessional and multisite development of the tool may have helped to better meet the needs of the PACU workflow. Other explanations include the 30‐min assessment interval, which left nurses enough time for other patient‐related activities, and the integration of the tool into the PDMS, which provided a guiding structure for the PACU workflow. Altogether, the user‐centred design, adaptation and implementation proved valuable [16]. Of note, already before the implementation of PANDA, nurses had reported very high confidence in their discharge decisions, reflecting the high training standards for PACU nurses in Switzerland [28, 29]. If the implementation of PANDA had negatively affected the nurses' satisfaction with the discharge decision, these differences would have been shown in our study.

We assumed the PACU to be a bottleneck in the postoperative workflow, and therefore proposed the PANDA tool could reduce OR holds, as shown previously [26]. However, we found a surprisingly low number of OR holds, which made it impossible to demonstrate any statistically relevant effects. Although the control period (August) and the PANDA period (late October to November) represent different calendar intervals, both were routine non‐holiday periods with comparable OR utilisation and similar numbers of OR and PACU cases. We therefore consider major seasonal effects unlikely. However, residual temporal or organisational differences between these periods cannot be fully excluded. The prevention of OR holds requires multidisciplinary communication and good knowledge of the bed capacity throughout all areas [23]. It may have been the case in our study that the OR and PACU teams had anticipated potential OR holds and prevented more events in both the pre‐ and post‐implementation periods.

A number of patients (13%) did not reach discharge readiness within 150 min and required a physician's decision to be discharged. Almost all of these patients had undergone general anaesthesia and about half of them were classified with an ASA‐PS score of 3 and higher. This finding is consistent with the literature, which confirms both variables as predictors for prolonged PACU stays [30]. The proportion of around 10% of patients who need clinical decisions beyond the frame of a discharge tool appears acceptable. Furthermore, it seems essential that a discharge tool not cover the more complex patients, as clinical decisions about these patients should be left to physicians.

## 5 Limitations

Our study has some limitations. First, this was a single‐centre study, which remains prone to bias, for example, by including patients who had undergone major surgery but not those who had undergone elective orthopaedic surgery. Second, there is considerable potential for selection bias in the nurses' surveys, as the response rate was only 50%. Additionally, it was not possible to evaluate the downstream effects of the tool, such as patient safety or workflow changes on peripheral wards. For these outcomes, larger studies with more sophisticated designs are needed. Additionally, matching on ASA physical status likely accounts for the complexity of patient comorbidities, it may be even better to match cases on actual patient comorbidities. However, a more differentiated matching was not possible in the PDMS system used, and the high sample size of the study likely compensated for this potential effect. Lastly, the survey results may be subject to selection bias given the limited response rate. Future prospective studies with larger samples are needed to confirm its effectiveness and generalisability.

## 6 Implication for Practice and Further Research

Our sub‐analysis revealed an LOS reduction for all surgical disciplines, however, not all of them were statistically significant. However, the effect of PANDA was more pronounced in disciplines with a longer median LOS (e.g., general surgery, kidney transplantation). Future studies with a prospective study design and larger sample sizes would be necessary to provide a detailed analysis of these differences. However, the differences in PACU LOS should be considered for the composition of the case‐mix to improve prospective OR and PACU capacity planning as the use of a structured discharge tool may support teams in anticipating resource needs more accurately [31]. Given the retrospective study design and the small staff sample size, the results should be interpreted with caution. Nevertheless, the findings suggest that the PANDA tool may support workflow standardisation and patient flow. Machine learning could potentially improve the scheduling/regulation/navigation of the OR and PACU capacity [32].

## 7 Conclusion

Implementing the semi‐automated PANDA discharge tool significantly decreased PACU LOS. In addition, PACU nurses reported high acceptance, usefulness and feasibility of the tool. Therefore, the PANDA discharge tool may optimise routine clinical practice to streamline PACU workflows, support resource allocation and decision‐making and promote standardisation. Reducing PACU LOS may also improve patient flow and capacity planning in high‐volume settings.

## Funding

The authors have nothing to report.

## Ethics Statement

The study was approved by the Cantonal Ethics Committee, Zurich, Switzerland and issued a declaration of no objection (Swissethics Request number: 2021‐00806, on 20 July 2021, Chairperson: P. Kleist). The reporting of this study adheres to the STROBE guidelines.

## Consent

Written informed consent was obtained from individuals who agreed to participate in the survey among nurses with the Informed Voluntary Consent Form.

## Conflicts of Interest

None of the authors declare Conflicts of Interest except DRS.

Declaration of CoI by DRS: Dr. Spahn is chair of the ABC‐Trauma Faculty, sponsored by unrestricted educational grants from Alexion Pharma Germany GmbH, Munich, Germany, CSL Behring GmbH, Marburg, Germany, and LFB Biomédicaments, Courtaboeuf Cedex, France. Dr. Spahn is also the president of Alliance Rouge, Bern, Switzerland, CEO of Swiss‐PBM‐Consulting GmbH, Zurich, Switzerland and a member of the Advisory Board of Saipient AG, Zurich, Switzerland.

Dr. Spahn received honoraria/travel support for consulting or lecturing from: Alliance Rouge, Bern, Switzerland, European Society of Anesthesiology and Intensive Care, Brussels, BE, Korean Society of Anesthesiologists, Seoul, Korea, Network for the Advancement of Patient Blood Management, Haemostasis and Thrombosis, Paris, France, Society for the Advancement of Blood Management, Mount Royal NJ, Alexion Pharmaceuticals Inc., Boston, MA, AstraZeneca AG, Baar, Switzerland, Baxter AG, Glattpark, Switzerland, Bayer AG, Zürich, Switzerland, B. Braun Melsungen AG, Melsungen, Germany, CSL Behring GmbH, Hattersheim am Main, Germany and Berne, Switzerland, CSL Vifor (Switzerland) Villars‐sur‐Glâne, Switzerland, CSL Vifor (International), St. Gallen, Switzerland, Haemonetics, Braintree, MA, USA, iSEP, Nantes, France, Novo Nordisk Health Care AG, Zurich, Switzerland, Octapharma AG, Lachen, Switzerland, Pharmacosmos A/S, Holbaek, Denmark, Werfen, Bedford, MA.

## Supporting information


**Figure S1:** Screenshot of the post‐anaesthesia discharge assessment (PANDA) in the patient data management system (PDMS). This is the user‐view during the assessment of patients in the postanaesthesia care unit (PACU). On the left side, nurses can rate the items with their criteria ‘yes’ (achieved) or ‘no’ (not achieved). The first four items are rated automatically by the PDMS using vital signs from patient monitor updated and stored every minute. The upper right side provides the PANDA algorithm. The lower right side shows the cumulative score of the PANDA with the discharge decision appearing green when all items are achieved. Special cases can be noted as well, for example, when a patient is discharged by a physician without achieving the PANDA criteria.

**Figure S2:** The figure shows the absolute standardised mean differences between the groups for each matching variable. The white dots (All) represent the absolute standardised mean differences of the total sample before matching (*n* = 8475). The black dots (matched) represent the absolute standardised mean differences after the sample was matched to the PANDA group (*n* = 4509) and the control group (*n* = 3966).

**Table S1:** Differences of PACU LOS in minutes between different types of surgery in the PANDA group compared to the control group. The values are estimated coefficients based on a weighted linear model with additional adjustment for surgery type, with plastic surgery being the reference category.

## Data Availability

Data available on request due to privacy/ethical restrictions.
