# Supplementary material for: Effectiveness of the Semi‐Automated Post‐ANaesthesia Discharge Assessment Tool: A Pre‐Post Study Using Propensity Score Matching
Source: Nurs Crit Care. 2026 Feb 11;31(2):e70393. doi: 10.1111/nicc.70393 (PMC12894805; doi:10.1111/nicc.70393)

**Figure S1:** Screenshot of the Postanaesthesia Discharge Assessment (PANDA) in the Patient Data Management System (PDMS). This is the user-view during the assessment of patients in the post anaesthesia care unit (PACU). On the left side, nurses can rate the items with their criteria ‘yes’ (achieved) or ‘no’ (not achieved). The first four items are rated automatically by the PDMS using vital signs from patient monitor updated and stored every minute. The upper right side provides the PANDA algorithm. The lower right side shows the cumulative score of the PANDA with the discharge decision appearing green when all items are achieved. Special cases can be noted as well, e.g. when a patient is discharged by a physician without achieving the PANDA criteria.


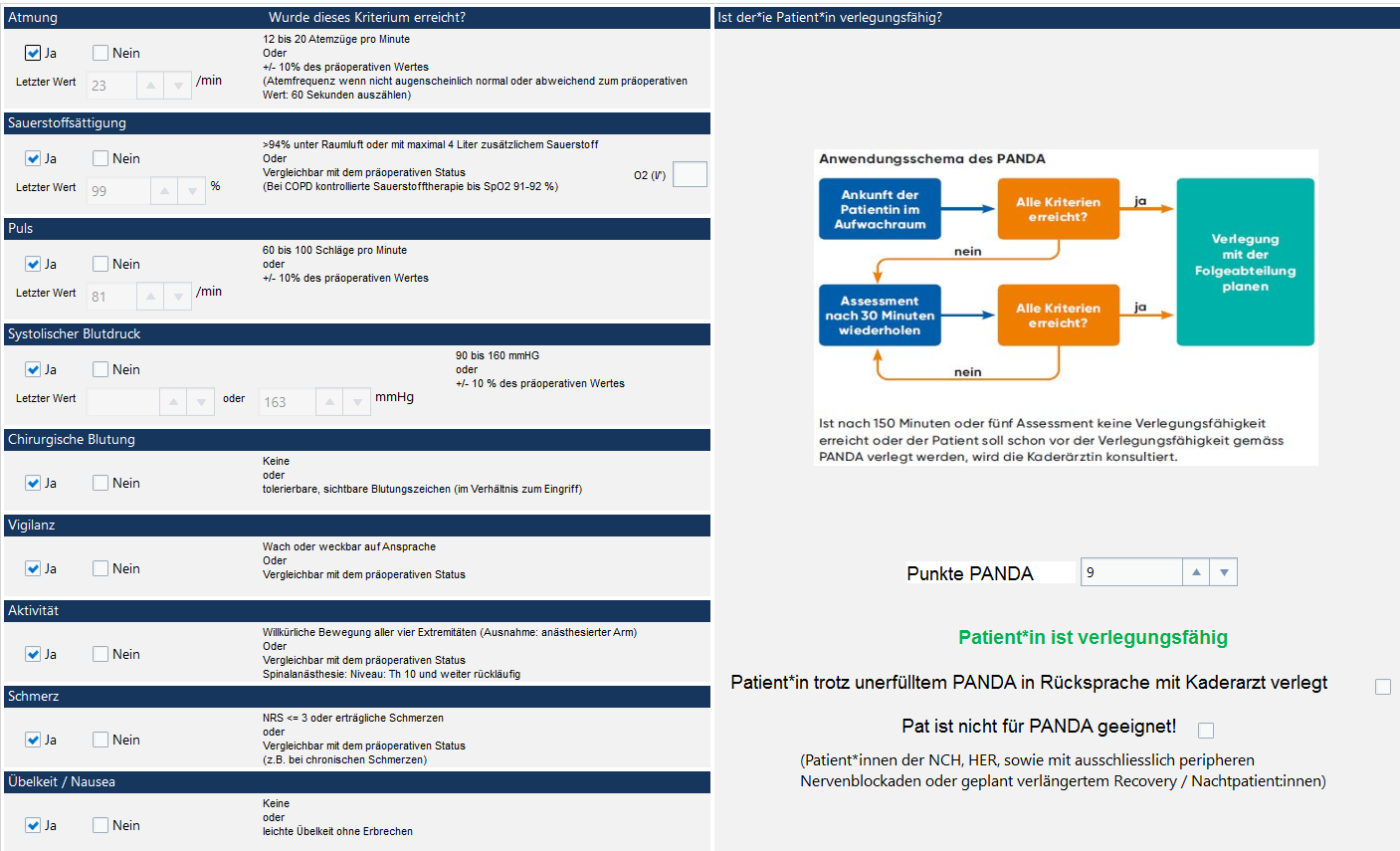

Supplement: Supplementary file 1 — Figure S1: Screenshot of the post‐anaesthesia discharge assessment (PANDA) in the patient data management system (PDMS). This is the user‐view during the assessment of patients in the postanaesthesia care unit (PACU). On the left side, nurses can rate the items with their criteria ‘yes’ (achieved) or ‘no’ (not achieved). The first four items are rated automatically by the PDMS using vital signs from patient monitor updated and stored every minute. The upper right side provides the PANDA algorithm. The lower right side shows the cumulative score of the PANDA with the discharge decision appearing green when all items are achieved. Special cases can be noted as well, for example, when a patient is discharged by a physician without achieving the PANDA criteria. [file NICC-31-0-s002.docx]
