# Supplementary material for: Effectiveness of the Semi‐Automated Post‐ANaesthesia Discharge Assessment Tool: A Pre‐Post Study Using Propensity Score Matching
Source: Nurs Crit Care. 2026 Feb 11;31(2):e70393. doi: 10.1111/nicc.70393 (PMC12894805; doi:10.1111/nicc.70393)

**Figure S2:** The figure shows the absolute standardized mean differences between the groups for each matching variable. The white dots (All) represent the absolute standardized mean differences of the total sample before matching (n=8475). The black dots (matched) represent the absolute standardized mean differences after the sample was matched to the PANDA group (n=4509) and the control group (n=3966).


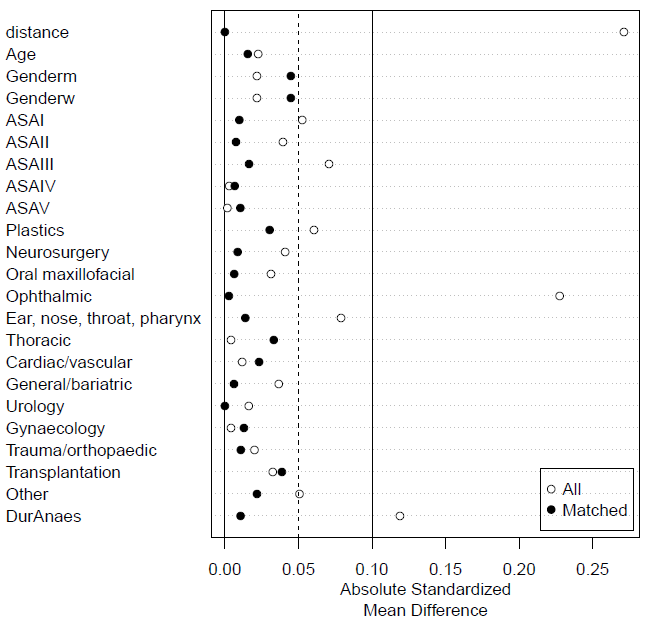

Supplement: Supplementary file 2 — Figure S2: The figure shows the absolute standardised mean differences between the groups for each matching variable. The white dots (All) represent the absolute standardised mean differences of the total sample before matching (n = 8475). The black dots (matched) represent the absolute standardised mean differences after the sample was matched to the PANDA group (n = 4509) and the control group (n = 3966). [file NICC-31-0-s001.docx]
