# Supplementary material for: Effectiveness of the Semi‐Automated Post‐ANaesthesia Discharge Assessment Tool: A Pre‐Post Study Using Propensity Score Matching
Source: Nurs Crit Care. 2026 Feb 11;31(2):e70393. doi: 10.1111/nicc.70393 (PMC12894805; doi:10.1111/nicc.70393)
Supplement: Supplementary file 3 — Table S1: Differences of PACU LOS in minutes between different types of surgery in the PANDA group compared to the control group. The values are estimated coefficients based on a weighted linear model with additional adjustment for surgery type, with plastic surgery being the reference category. [file NICC-31-0-s003.docx]

**Table S1:** Differences of PACU LOS in minutes between different types of surgery in the PANDA group compared to the control group. The values are estimated coefficients based on a weighted linear model with additional adjustment for surgery type, with plastic surgery being the reference category.

| **Characteristics** | **Coefficient** | **95% CI** | **p-value** |
| --- | --- | --- | --- |
| PANDA group (overall) | -16 | -20, -12 | <0.001 |
| Ophthalmic surgery | -23 | -32, -14 | <0.001 |
| Urologic surgery | -13 | -21, -4.4 | 0.003 |
| Ear, nose, throat, pharynx surgery | -12 | -19, -5.8 | <0.001 |
| Gynaecologic surgery | -12 | -19, -4.4 | 0.001 |
| Oral maxillofacial surgery | -5.0 | -15, 5.4 | 0.3 |
| Plastic surgery | - | - | - |
| Thoracic surgery | 12 | -5.7, 30 | 0.2 |
| Trauma / Orthopaedic surgery | 23 | 15, 31 | <0.001 |
| General and bariatric surgery | 24 | 16,32 | <0.001 |
| Cardiac /Vascular surgery | 37 | 25, 49 | <0.001 |
| Neurosurgery | 40 | 26, 53 | <0.001 |
| Other | 88 | 58,118 | <0.001 |
| Kidney Transplantation surgery | 206 | 173, 239 | <0.001 |

Abbreviations: PANDA: Postanaesthesia Discharge Assessment, PACU: post anaesthesia care unit
